# Automatic Artifact Removal in EEG of Normal and Demented Individuals Using ICA–WT during Working Memory Tasks

**DOI:** 10.3390/s17061326

**Published:** 2017-06-08

**Authors:** Noor Kamal Al-Qazzaz, Sawal Hamid Bin Mohd Ali, Siti Anom Ahmad, Mohd Shabiul Islam, Javier Escudero

**Affiliations:** 1 Department of Electrical, Electronic & Systems Engineering, Faculty of Engineering & Built Environment, Universiti Kebangsaan Malaysia, UKM Bangi, Selangor 43600, Malaysia; sawal@ukm.edu.my; 2 Department of Biomedical Engineering, Al-Khwarizmi College of Engineering, Baghdad University, Baghdad 47146, Iraq; 3 Department of Electrical and Electronic Engineering, Faculty of Engineering, Universiti Putra Malaysia, UPM Serdang, Selangor 43400, Malaysia; sanom@upm.edu.my; 4 Malaysian Research Institute of Ageing (MyAgeing), Universiti Putra Malaysia, Serdang, Selangor 43400, Malaysia; 5 Faculty of Engineering, Multimedia Universiti, MMU Cyberjaya, Selangor 63100, Malaysia; shabiul@ukm.edu.my; 6 Institute for Digital Communications, School of Engineering, The University of Edinburgh, Edinburgh EH9 3FB, UK; javier.escudero@ed.ac.uk

**Keywords:** electroencephalography, independent component analysis, wavelet, spectral analysis, vascular dementia, mild cognitive impairment

## Abstract

Characterizing dementia is a global challenge in supporting personalized health care. The electroencephalogram (EEG) is a promising tool to support the diagnosis and evaluation of abnormalities in the human brain. The EEG sensors record the brain activity directly with excellent time resolution. In this study, EEG sensor with 19 electrodes were used to test the background activities of the brains of five vascular dementia (VaD), 15 stroke-related patients with mild cognitive impairment (MCI), and 15 healthy subjects during a working memory (WM) task. The objective of this study is twofold. First, it aims to enhance the recorded EEG signals using a novel technique that combines automatic independent component analysis (AICA) and wavelet transform (WT), that is, the AICA–WT technique; second, it aims to extract and investigate the spectral features that characterize the post-stroke dementia patients compared to the control subjects. The proposed AICA–WT technique is a four-stage approach. In the first stage, the independent components (ICs) were estimated. In the second stage, three-step artifact identification metrics were applied to detect the artifactual components. The components identified as artifacts were marked as critical and denoised through DWT in the third stage. In the fourth stage, the corrected ICs were reconstructed to obtain artifact-free EEG signals. The performance of the proposed AICA–WT technique was compared with those of two other techniques based on AICA and WT denoising methods using cross-correlation XCorr and peak signal to noise ratio (PSNR) (ANOVA, p ˂ 0.05). The AICA–WT technique exhibited the best artifact removal performance. The assumption that there would be a deceleration of EEG dominant frequencies in VaD and MCI patients compared with control subjects was assessed with AICA–WT (ANOVA, p ˂ 0.05). Therefore, this study may provide information on post-stroke dementia particularly VaD and stroke-related MCI patients through spectral analysis of EEG background activities that can help to provide useful diagnostic indexes by using EEG signal processing.

## 1. Introduction

EEG sensors provide a non-invasive method to measure the electrical activity of the brain by placing electrodes over the scalp. This sensing technology is non-invasive and the EEG can achieve high temporal resolution to reflect the dynamics of brain activity directly. The EEG has been widely used for both medical diagnosis and neurobiological research [1]. Therefore, like EEG sensors which offers a quantitative approach to the detection of possible parameters that could indicate how severe dementia is. EEG tracks information processing with milliseconds precision and has been subjected to interpretation by clinician visual inspection that results in acceptable and successful diagnosis results. The first EEG clinical observation was illustrated by Berger at the beginning of the last century [2]. Berger made the unexpected observation that when observers opened their eyes, the EEG oscillations in the Berger rhythm decreased in amplitude or disappeared completely. In general, the amplitude and frequency range of clinical EEG waveforms are 10–70 μv and 1–100 Hz, respectively. Furthermore, important data are provided by EEG waveforms, which are separated into five frequency bands, namely, delta (δ), theta (θ), alpha (α), beta (β), and gamma (γ) [3,4]. In the context of physiology, the power distribution of various frequency bands may be determined based on EEG signal characterization. Hence, the determination of important data regarding cognitive function and memory performance may depend crucially on EEG relative powers [5].

Cognitive impairment after stroke is common and introduces individuals to the vascular cognitive impairment (VCI) spectrum. The VCI spectrum can be viewed as consequences in the cognitive domain starting from mild cognitive impairment (MCI) to severe dementia [6].

MCI refers to the decline in cognitive function that is greater than expected with regard to the age and education level of an individual. Nevertheless, the reduced cognitive function does not interfere with daily activities. Clinically, MCI is the transitional stage between early normal cognition and late severe dementia. MCI is considered heterogeneous because some MCI patients develop dementia, whereas others stay as MCI patients for many years. However, patients who were diagnosed with MCI exhibit a high risk of developing dementia, and this risk is thrice that of people without cognitive dysfunction. The most commonly observed symptoms of MCI are limited to memory problems, but the daily activities of patients remain the same [7].

Post-stroke dementia is associated with vascular and neurodegenerative changes, neuronal dysfunctions, and neuronal deaths [8]. Cognitive impairment and dementia following stroke diagnosis may involve multiple functions, including attention, memory, language, and orientation [9,10]. The highest effect of a stroke at the time of diagnosis is observed on the attention, executive functions, and memory.

Vascular dementia (VaD) is anticipated to be developed by around 30% of individuals who survived a stroke within twelve months following stroke diagnosis [11]. From a clinical perspective, the cognitive disorder called mild cognitive impairment (MCI) is surprisingly pervasive and is deemed to be an intermediary between normal cognitive function for old age and severe dementia [12].

Stroke mostly affects the attention and executive function, which are associated with working memory (WM). Thus, WM was considered in this study. WM is the ability to provide a temporary storage and to manipulate information for complex cognitive tasks such as attention, comprehension, reasoning, planning, and learning within a short period (10–15 s up to 60 s) [13]. According to the WM capacity of an individual, WM is considered a temporary storage system with a capacity of 7 ± 2 items [13,14].

The memory system of the human brain is a complex structure with different functionalities. This system refers to the process of how our brain transmits and stores available information for future usage, with or without conscious awareness. In this regard, three types of memory processes, namely, sensory memory, short-term memory, and long-term memory, can be distinguished [15].

The sensory memory is an ultra-short-term memory that decomposes shortly (200–500 ms) after the perception of an item. The sensory memory passes information to short-term memory by selecting the presently intended information through attention. Short-term memory normally expires within 10 s to 15 s, but a longer retention (up to 60 s) is possible when necessary.

WM is a short-term memory that can maintain and manipulate information for brief periods. On the basis of an individual’s memory capacity, WM is considered as a temporary memory that can store approximately 7 ± 2 items for a short period (60 s) [13,14].

Finally, long-term memory is the information stored in the brain and retrievable over a long period (often over the entire life span of the individual); it involves a process of physical changes in the structure of neurons in the brain [14].

Researchers have studied EEG signals to identify brain changes associated with the cognitive function and memory performance [16,17,18,19]. For instance, Klimesch and other researchers [5,20,21,22] have identified the pattern in the brain activity during WM tasks, these changes can be summarized by increasing θ and γ powers during WM load and decreasing α magnitude and α/β ratio as WM load increased.

The documented wave activities may be distorted by the fact that various kinds of artifacts could interfere with the EEG signals [23,24]. These artifacts affect examinations of EEG signals as they are typically capable of imitating and/or superimpose brain pathological activity [1]. Furthermore, EEG frequencies can become overlapped by the main artifacts interfering with EEG signals, such as ocular artifacts (OAs) (e.g., eye blinks and movements), cardiac artifacts (CAs), muscle activities (MAs), and power line interference noise [23,24,25].

From the clinical perspective, several methods have been applied to address artifacts that influenced EEG recording to interpret EEG for pathological activities accurately. Therefore, studies on artifact removal have been proposed, including epoch rejection [26], regression techniques [27], and blind source separation (BSS) [28,29].

Epoch rejection is frequently considered the simplest method to eliminate artifacts based on the visual inspection of recorded EEG signals. Rejecting epochs is unacceptable in real-time applications. Rejecting epochs are unacceptable in real-time applications. Discarding task-relevant neural responses may cause insufficient the EEG data makes this method ineffective in many neural studies [30,31]. Epoch rejection causes loss in raw data with widespread artifactual contamination and is highly time-consuming [32,33].

In regression in the time domain, the artifact is subtracted from each scalp electrode. The performance of this method is affected by the bidirectional contamination problem; hence, the recorded data typically exhibit a common cerebral pattern, which leads to their partial removal via the regression method [33,34].

Many researchers have used independent component analysis (ICA) to separate distinct artifacts from EEG signals efficiently [35,36]. ICA is used essentially to extract and separate sources that underlie the multi-channel measurements of biomedical signals into their constituent components. The success of ICA in biomedicine relies on the fulfillment of several conditions, such as the sources being statistically independent and having non-Gaussian distributions, and the mixtures being a linear combination of the independent sources [37]. Most available studies have used visual inspection and manual artifact extraction, which are time-consuming, inconvenient when dealing with a large amount of EEG data, unsuitable for real-time processing, and subject to human bias [38,39]. To overcome these problems, several studies on the automatic identification of artifacts have been developed using ICA [40,41,42]. Zhou et al. proposed and evaluated the use of ICA to automatically remove eye movement artifacts from the EEG [43,44], Romero et al. proposed a fully automatic procedure for ocular correction from spontaneous EEG signals based on blind source separation (BSS) [45,46], Vázquez et al. [40] successfully proposed an automated system to reject a good proportion of artifactual components extracted by ICA while preserving EEG components. Radüntz et al. [41] presented a method that would automatically select artifact components based on the map of scalp topographies. Sameni et al. [42] applied the ICA algorithm to remove electrooculogram (EOG) artifacts from multichannel EEG recordings. However, ICA may result in the loss of residual EEG information because the corresponding signal of interest and noise overlap in the time-frequency and spatiotemporal domains.

Meanwhile, wavelet transform (WT) denoising technique is widely used in non-stationary biomedical signals processing due to its localization characteristics in both the time and frequency domains [47,48]. WT has also been employed in EEG signal analysis because it can remove electromyogram (EMG) and EOG noise [49,50]. Discrete WT (DWT) has also been used to decompose the EEG signals into the frequency sub-bands [51,52].

Recently, using a combination of denoising methods from EEG has gained attention for multi-channel processing [38,39,53]. Castellanos et al. [53] proposed the WT-enhanced ICA method by applying a WT threshold to the decomposed ICs. WT thresholding allows artifactual components identification in time–frequency domains in order to reconstruct the brain activity that has imbedded into these components. Accordingly, the previous identification of artifactual ICs is unnecessary, and all the ICs are WT denoised [53]. Ghandeharion et al. [54] presented a fully automatic method for OA suppression using WT and ICA. WT was used to enhance the detection of artifactual ICs, particularly for OA suppression. Meanwhile, Castellanos et al. [53] used wavelet to enhance artifact suppression. Akhtar et al. applied the concept of spatially constructed ICA to extract noisy ICs, and then WT denoising was performed on the components extracted from ICA to remove OA [38]. Similarly, Klados et al. [55] used a regression technique to denoise ICs related to OAs. In addition, Mammone et al. [39] inverted the procedure. In particular, they used DWT to decompose each channel of the recorded EEG into the four bands of EEG signals. Each channel was represented by four WT components, and the artifactual ICs were automatically identified by WT to be passed into ICA. Artifactual ICs were rejected before ICA reconstruction, inverse ICA (inv–ICA), followed by inverse DWT (IDWT) [39]. The concept behind these hybrid techniques is to filter brain informative data while reducing the loss of cerebral activity information, which may be embedded into the artifact components and will be lost by rejecting contaminated ICs. In this manner, EEG activity is mostly preserved.

However, in nearly all of the previous studies that used ICA, the ICs marked as artifactual components were either manually or automatically identified to be rejected and the other ICs were used to reconstruct clean EEG data. If some brain activities are imbedded to artifactual ICs, then rejecting these ICs will result in the loss of desired information.

Thus, to address the aforementioned problems, in this study, an AICA–WT technique has been proposed to denoise critically marked ICs using DWT and to reconstruct ICA-corrected EEG signals. Therefore, the advantage of the proposed technique is to detect and extract artifactual components using higher-order statistics along with entropy as markers for automatic artifact discrimination. Accordingly, the extracted features and separation between dementia patients and control subjects will be improved.

## 2. Methods and Materials

### 2.1. Methods

**Figure 1:**
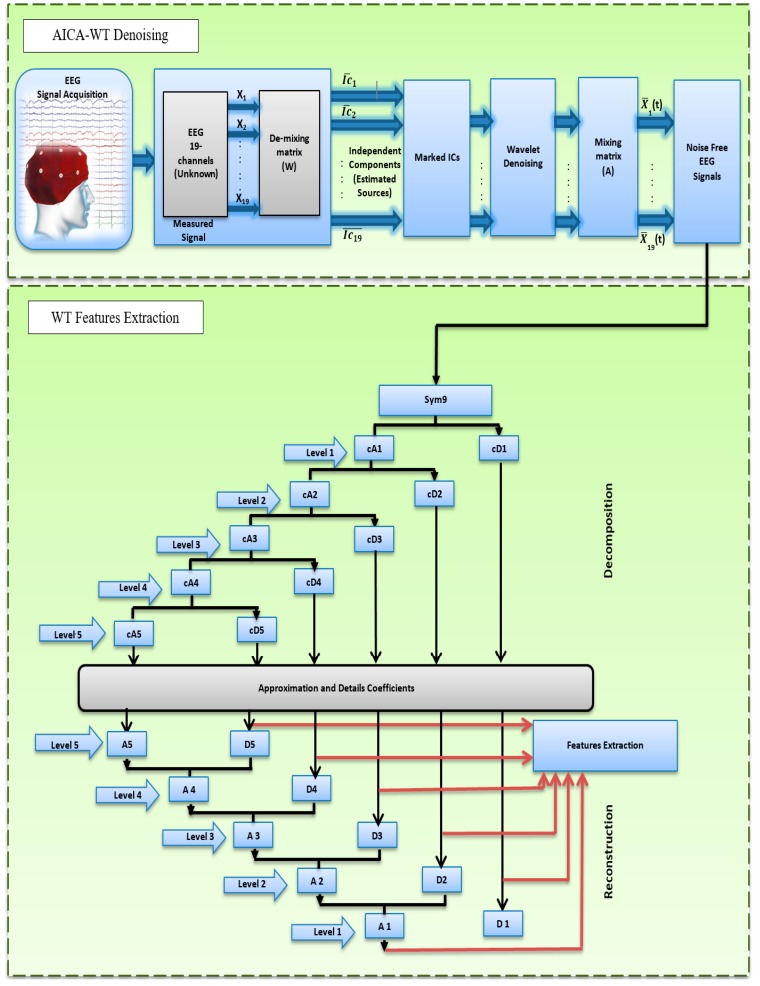
The proposed block diagram of this study.

Figure 1 shows the general block diagram of this study. This study has two objectives. First, it aims to enhance the recorded EEG signals using a novel AICA–WT technique; second, to investigate the spectral features that characterize the dementia patients compared to the control subjects using EEG bands that were extracted through DWT decomposition from artifact-free signals.

### 2.2. Subjects and EEG Recording

A NicoletOne (V32) from the manufacturer VIASYS Healthcare Inc. (Cullman, AL, USA), was employed to obtain the sets of EEG data. The number of electrodes used was nineteen, of which one was a ground electrode and two were system reference electrodes. In keeping with the 10–20 international system, EEG signals from locations Fp2, F8, T4, T6, O2, Fp1, F7, T3, T5, O1, F4, C4, P3, F3, C3, P3, Fz, Cz, and Pz were recorded with a forehead ground electrode and a referential montage with ipsilateral ear references. A series of hardware low pass, high pass and notch filters were incorporated in the EEG device. A frequency value of 0.3 Hz was attributed to the low pass filter equivalent to 3 dB; frequencies of 70 and 50 Hz were respectively established for the upper cutoff and the notch filter; according to the application, the frequency value of sampling was 256 Hz. Furthermore, the electrode impedance was less than 10 kilo-ohms. Precision was enhanced through digitalization of the signal based on 100 μv/cm sensitivity and 12 bit A/D converter.

The present study involved the analysis of the EEG data sets of 35 subjects, of which 15 were healthy subjects, 15 were subjects with stroke-related MCI, and 5 were subjects with VaD. One-way ANOVA has been performed, the p value of 0.435 illustrates an indication that there is insignificant difference among the ages of the three populations (p > 0.05). The stroke-related MCI and VaD groups were respectively selected from the stroke unit of the Pusat Perubatan Universiti Kebangsaan Malaysia (PPUKM) and from the PPUKM Neurology Clinic. The criteria of the National Institutes of Health Stroke Scale (NIHSS) were met by the post-stroke subjects [56]. Magnetic resonance imaging (MRI) or computed tomography (CT) enabled subjects’ brain to be scanned and the diagnosis of every subject was undertaken on the basis of medical history as well as clinical and laboratory investigations. No earlier mental irregularities had been experienced by the healthy subjects. Furthermore, neuropsychological tests, such as Mini-Mental State Examination (MMSE) [57] and Montreal Cognitive Assessment (MoCA), were applied to the subjects [58]. The study demographic data and the results obtained by each of the three groups of subjects on the neuropsychological tests are presented in Table 1.

**Figure 2:**
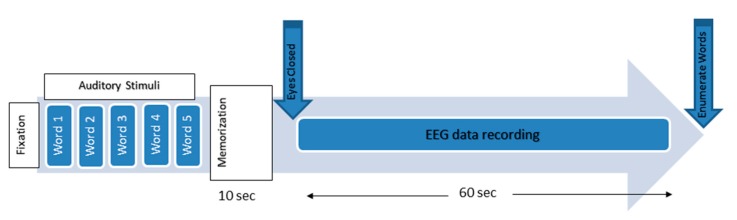
The working memory experimental paradigm [59].

In this EEG study, an auditory WM task session was conducted in this study. All experiment protocols and recording procedures were approved by the Human Ethics Committee of the PPUKM. All volunteers signed informed consent forms (ICF). The session started with a 0.5 s fixation cue when the subjects were asked to be motionless as much as possible. A simple WM task was then performed, during which the subjects were asked to memorize five words for 10 s. Afterward, they were asked to remember these words with their eyes closed, and the EEG data were recorded. After 60 s, the patients were asked to open their eyes and enumerate all words that they could remember [12]. (Figure 2).

MMSE is a brief test of 30-point questionnaire that is used extensively in clinical and research settings to measure cognitive impairment. It is commonly used in medicine and allied health to screen for dementia. MoCA test is a 30-point test and it considered as a promising tool for detecting MCI and Early AD [60,61,62]. In this study, MMSE has been used with MoCA to detect the early stages of dementia.

The scores of the patients in the WM task were included in the MMSE and MoCA (for the attention and concentration parts, respectively). These scores were computed based on the number of remembered words. To be included in this study, the control subjects should remember or enumerate all words at the end of the EEG recording and should obtain the maximum score in the attention and concentration parts of the MMSE and MoCA assessments.

### 2.3. AICA–WT Technique Methodology

In this study, the AICA–WT technique is proposed and discussed as a fully automatic hybrid technique. This technique is used to combine the positive aspects of both ICA and DWT and to control some of their shortcomings. AICA–WT is used to improve the recorded EEG signals. To detect and remove OAs, CAs, and MAs from EEG data, the AICA–WT technique has been used as a four-stage approach. In the first stage, the ICs were estimated. In the second stage, three-step artifact detection metrics based on the calculation of kurtosis (Kurt), skewness (Skw), and sample entropy (SampEn) are applied. These metrics are used to detect the aforementioned artifacts. Therefore, to remove artifacts automatically and save computational time and complexity, only the components identified as artifacts are marked as critical and arranged into new dataset to be denoised through DWT in the third stage. In the fourth and final stage, the inv-ICA are performed to obtain denoised (artifact-free) EEG signals. The four main stages will be discussed in the following sections.

#### 2.3.1. Linear Mixing Model and ICA Algorithm

**Figure 3:**
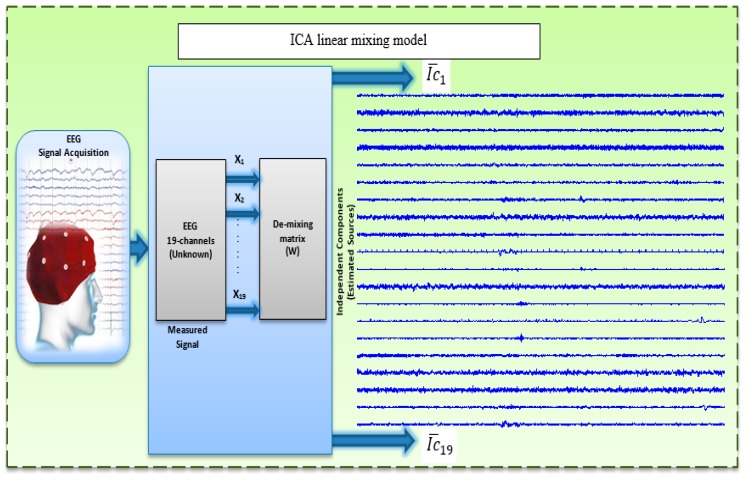
Linear mixing model.

ICA is a powerful statistical method for separating mixed signals based on several assumptions. The most important assumption is that existing sources are statistically independent from one another. The mixing process should also be linear and instantaneous [63,64]. Researchers have proven that EEG data fulfill these hypotheses [28,65]. The aim of ICA is to estimate the set of *n* unknown components, $s\left(t\right)=\left[s_{1}\left(t\right),\ldots ,\;s_{n}\left(t\right)\;\right]$, which were linearly mixed by the matrix A, the ICA linear transform equation is:

(1) x(t)=As(t)

where x(t) represents the EEGs and x(t) and s(t) are supposed to have zero mean. The ICA use the the higher-order statistics of x(t) to compute the demixing matrix W, which is the inverse matrix of A to be linearly represented the independent components. Then, under such assumptions, the ICs can be estimated by Equation (2) [28,63,66]:

(2) y(t)=Wx(t)

where $y\left(t\right)=\left[y_{1}\left(t\right),\ldots ,\;y_{n}\left(t\right)\;\right]$ is the vector that estimate the ICs (Figure 3).

Accordingly, ICA is considered a powerful technique for finding artifact components and brain activity components that may be affected more by dementia than other components [67,68]. In this study, the aim was not to isolate specific physiological activities but to denoise the EEG of normal and demented individuals to enhance features during WM. A criterion must be established to compare ICs from different EEG epochs and subjects as well as to determine which components are sensitive to noise [67,68,69]. Numerous algorithms can be employed to decomposed the ICs such as fast ICA (FastICA) [70], Information Maximisation (InfoMax) [35] and Joint Approximate Diagonalization of Engine Matrices (JADE) [71]. In this study, EEG signals were decomposed using the FastICA algorithm based on the fixed-point algorithm proposed by Hyvärinen [70]. The FastICA algorithm was used because of its simplicity, fast convergence, and efficiency to decompose the recorded EEG and to extract the new component matrix $\hat{s}$, wherein the artifacts were OAs, such as eye blinks and movements, CAs, and transient strong MAs [72].

#### 2.3.2. Artifact Detection Metrics

**Figure 4:**
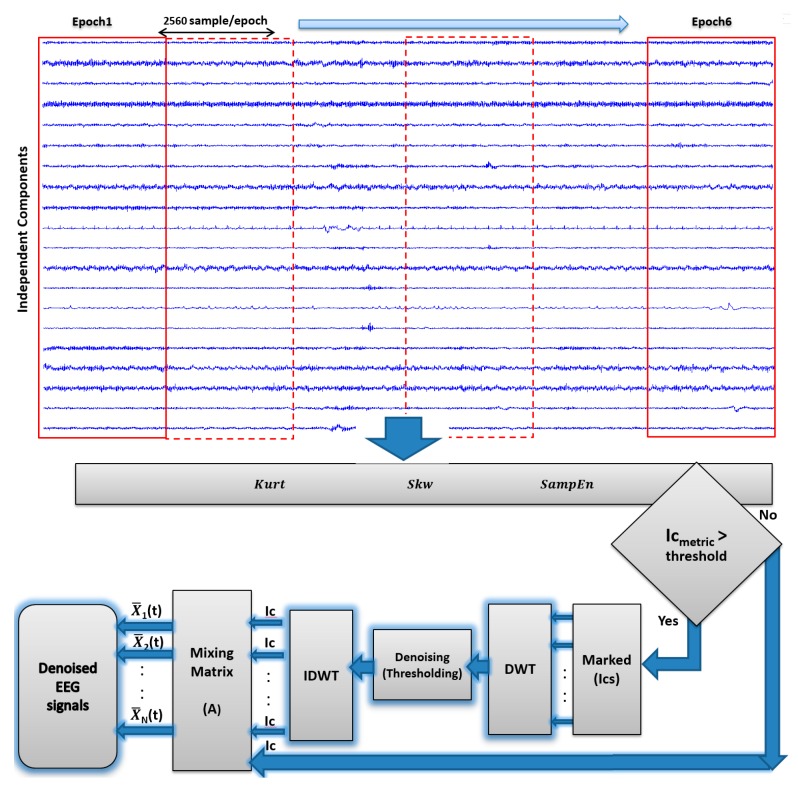
AICA–WT denoising technique.

Noise factors determine how reliable EEG signals are. During EEG recordings, artifacts usually originate from OAs like eye blinks and movements [73]. In general, OAs showed the higher amplitude and lower frequency than the recorded EEG signals of interest, the OA activity originate mainly from the frontal regions of the scalp within the frequency range of less than 5 Hz [74]. Saccade movements, which are caused by ocular muscles, generate MAs [73]. Transient strong MAs are generated from patient movements and can be picked up on the scalp; MA signal frequencies are concentrated within the frequency of greater than 30 Hz [39,75]. Moreover, by comparison to the recorded brain activity, the strength of the magnetic field generated by CAs in the 0–40 Hz frequency range is greater [76]. Given such changes in the artifact-contaminated EEG signals, ICA is committed to find linear components that are both statistically independent and non-Gaussian [77]. Accordingly, it is important to treat and remove artifacts from the EEG signals carefully, to avoid incorrect results and conclusions [77]. In line with this, to improve result accuracy, artifacts must be eliminated from EEG signals with care. ICs approximated with FastICA [70] were separated into six epochs of ten seconds without overlap, each epoch being 2560 data points long (one segment). The peak and random characteristics of the artifacts were distinguished based on high statistical order using Kurt, Skw, and entropy using SampEn metrics. These metrics were separately computed for each epoch to mark the peakness and randomness of the artifacts separate computation of Kurt, Skw, and SampEn metrics for every epoch [32,64,78]. In the following part, the block diagram of the AICA–WT denoising technique is presented (Figure 4).

A. Skewness (Skw) and Kurtosis (Kurt)

Let $m_{n}=E\left\{{\left(x-E\left\{x\right\}\right)}^{n}\right\}$ be the nth central moment of the Skw and Kurt distributions. The Skw and Kurt are defined as in Equations (3) and (4) respectively:

(3) $$Skw=\frac{m_{3}}{{\left(m_{2}\right)}^{3/2}}$$

(4) $$Kurt=\frac{m_{4}}{{\left(m_{2}\right)}^{2}}-3$$

Skw is the normalized third-order moment of amplitude distribution. If the distribution is symmetrical, then Skw is zero. By contrast, large Skw values are associated with the asymmetry degree of amplitude distribution. Skw is used to detect CAs isolated in ICA components [66,76,78].

Kurt is the normalized fourth-order cumulant; it measures the non-Gaussianity peakness for ICA due to its computational and theoretical simplicity [39,79]. Kurt is used to recognize the distribution of highly peaky components, including transient strong MAs, CAs, and OAs [66,76]. Kurt is negative for “flatter” than Gaussian amplitude distributions, such as sub-Gaussian (platykurtic) distributions. By contrast, Kurt is positive for super-Gaussian (leptokurtic) distributions [66,76].

B. Sample Entropy (SampEn)

SampEn quantifies disorder or irregularity; high values are associated with numerous irregular signals [80,81]. SampEn is tested to detect activities such as CAs and OAs because their wave patterns are more regular compared with those of other activities [80,81]. SampEn is computed using the algorithm presented in [82], which is defined as:

(5) $$SampEn\left(m,r,N\right)=-\ln\left[\frac{A^{m}\left(r\right)}{B^{m}\left(r\right)}\right]$$

where N is the length of the EEG time series. For our analysis, SampEn is computed with a run length of epochs, m=2 and tolerance, r=0.2×SD [83].

To detect artifactual components, Kurt, Skw, and SampEn were tested for each IC. Then, they were normalized to zero-meanand unit variance corresponding to each IC. ICs with Kurt, Skw, and SampEn that exceeded the threshold of ±1.2 were marked as critical [39,64,76]. Moreover, if a component exceeded the threshold in over 20% of the epochs (at least 2 epochs), then the component was marked as critical. All marked components will not be rejected but they will be denoised using DWT. The practical value of the threshold is selected through trial and error. The threshold value of ±1.2 is not a drawback of AICA–WT proposed technique as the selected ICs will not be cancelled and they will be cascaded to WT to be denoised. Thus, large values of Kurt and Skw are related to leptokurtic and asymmetric IC components, which may be related to OAs and CAs [39,64]. Subsequently, the artifact identification procedure based on estimating Kurt, Skw, and SampEn is used to detect the aforementioned artifacts. Therefore, to remove artifacts automatically and save computational time and complexity, only the components that exceed a predefined threshold are marked as artifacts and arranged in a new artifactual dataset to be denoised through DWT. Wavelet enhanced marked ICs.

Artifacts in one or multiple channels are usually detected with ICA. However, this method fails to reveal artifact frequency as the signal is located in the time domain. This drawback can be solved by employing WT, whose capabilities of time and frequency domain localization are exceptional [39]. Thus, EEG signal pre-processing is enhanced with the AICA–WT method.

Wavelet (WT) has the ability in resolving EEG into specific time and frequency components with good localization in time at high frequencies and good localization in frequency at low frequencies. The discrete wavelet transform (DWT) is fast non-redundant transform used in practice for analyzing both the low and high frequency components in the EEG signals because it is less computational time than the continuous wavelet transform (CWT) [84]. The DWT can be processed by obtaining the discrete value of the parameters a and b, as in Equation (6). It can be obtained as a set of decomposition functions of the correlation between the signal f(t) and the shifting and dilating of one specific function called mother wavelet function ψ(t). Mother wavelet (MWT) is shifted by the location parameter (b) and dilated or contracted by frequency scaling parameter a, as in (Equation (7)) [85,86]:

(6) DWTm,n(f)=a0−m2∫​f(t)ψ(a0−mt−nb0)dt

$a_{0}$ and $b_{0}$ values are set to 2 and 1, respectively.

(7) $$\psi_{a,b}\left(t\right)=\frac{1}{\sqrt{a}}\psi \left(\frac{t-b}{a}\right),\;a\epsilon {\mathbb{R}}^{+},\;b\epsilon \mathbb{R}$$

Mallat developed a way of implementing DWT, the DWT provides a non-redundant representation of the signal and its values constitute the coefficients in a wavelet series. DWT decomposed the signal into different frequency bands by passing the signal through two quadrature mirror filters (QMF) at the different scales in term of finite impulse response (FIR), where the filter h is related to the scaling function, while filter g is related to the mother wavelet as given in Equations (8)–(10), for further technical details to some references [87,88,89]:

(8) $$g\left(h\right)={\left(-1\right)}^{n}h\left(1-n\right)$$

(9) $$\phi \left(x\right)=\sum_{n}h\left(n\right)\sqrt{2}\phi \left(2x-n\right)$$

(10) $$\psi \left(x\right)=\sum_{n}g\left(n\right)\sqrt{2}\;\phi \left(2x-n\right)$$

The QMF output is characterized as shown in Equations (11) and (12):

(11) $$H_{L}=\sum_{n}h\left(n-2L\right)x\left(n\right)$$

(12) $$G_{L}=\sum_{n}g\left(n-2L\right)x\left(n\right)$$

When the signal x(n) act as LPF it convolved with h(n−2L), otherwise it acts as HPF and convolved with g(n−2L). The result is transforming the original signal into two sub bands [0−FN2] and [FN2−FN]. It is significant that the $H_{L}$ is the approximation components (A) and it represents the lower resolution components, and $G_{L}$ is the details decomposition components (D) that describes the high resolution components [90,91].

Several parameters have to be selected carefully while using a DWT-based processing methods. These are the MWT basis function, the thresholding method and the WT decomposition level.

In this study, DWT using symlet MWT of order 9 “sym9” and SURE threshold were selected to be used [59]. The adaptive soft thresholding method, SURE threshold have been used. In SURE method the threshold value is achieved based on Stein’s unbiased risk estimation [92] and it used in [93,94,95]. The EEG signal was subjected to a five-level decomposition (the sampling frequency of this study was 256 Hz). After the threshold were applied for each level, the noises on the marked components in the artifactual datasets were removed. Then coefficients were reconstructed using inverse DWT (IDWT). The denoised components were returned back to the original components set.

#### 2.3.3. Reconstruction

Finally, the corrected ICs were reconstructed to become $\hat{x}$, the new data set which represents the ICA estimated of the original, but artifact free EEG data, as shown in Equation (13):

(13) $$\hat{x}\left(t\right)=A\hat{s}\left(t\right)$$

where $\hat{s}\left(t\right)$ the new component matrix.

### 2.4. WT Based Denoising and ICA Rejection

The WT and ICA methods have been widely used as denoising methods. In this study, DWT was applied as a denoising method with “sym9” and SURE threshold [59]. AICA rejection, that is, zeroing the artifactual ICs, was also performed. Notably, the proposed AICA–WT technique was used as a denoising tool by enhancing marked ICs using DWT. To conduct a comparison, cross-correlation XCorr and peak signal to noise ratio (PSNR) were performed between the recorded EEG and noise-free EEG using the AICA–WT, WT and AICA rejection techniques respectively. The correlation XCorr and PSNR between the EEG signal of interest x and the denoised EEG y is expressed in Equations (14) and (15) respectively [96,97]:

(14) $$XCorr\left(x,y\right)=\frac{\sum \left(x-\bar{x}\right)\left(y-\bar{y}\right)}{\sqrt{\sum {\left(x-\bar{x}\right)}^{2}{\left(y-\bar{y}\right)}^{2}}}$$

(15) $$PSNR=20\;\log\left[\frac{max\left[x\right]}{RMSE}\right]$$

where $\bar{x}$ and $\bar{y}$ are the mean of the recorded and noise-free EEG x and y, respectively, and N is the length of the selected window and RMSE is the root-mean-square error that can be calculated using Equation (16):

(16) $$RMSE=\sqrt{\frac{1}{N}\overset{N}{\underset{i=1}{\sum }}{\left(x-y\right)}^{2}}$$

### 2.5. Wavelet Decomposition

In order to extract the fundamental EEG power bands to perform the second fold of this study objective, DWT technique was also used as a decomposition method. WT analysis helps in quantifying the changes in EEG in a hierarchical scheme of nested sub-spaces called multi-resolution analysis (MRA). Therefore, DWT has been applied using “sym9” MWT [59] and a five-level decomposition through DWT. Six sub-bands decomposition coefficients were achieved from the EEG signal, particularly the decomposition detail coefficients (cD1 to cD5) and the decomposition approximation coefficient (cA5). The signals at each level were reconstructed using IDWT, where (D1 to D5) are the five reconstruction details and A5 is the reconstruction approximation of the sub-bands signal (see Table 2) [59].

### 2.6. Feature Extraction

Modifications in the spectra of the EEG data sets of the above-mentioned three groups of subjects were detected with the help of relative power (RP) features.

In this work, to quantify EEG changes during a WM task, RP features were calculated taking into account the frequency ranges shown in Table 2.

The sub-band WT-based features provided a representation of the denoised EEG signal. Therefore, a fourth-order Butterworth band pass filter was also applied to each α and β to extract the sub-bands of α and β. The EEG signals were then classified into five frequency bands. Each frequency band presents its own physiological significance [98] as follows: alpha1 ($\alpha_{1}$: 8 ≤ f ≤ 10.5) Hz, alpha2 ($\alpha_{2}$: 10.5 ≤ f ≤ 13) Hz, beta1 ($\beta_{1}$: 13 ≤ f ≤ 16) Hz, beta2 ($\beta_{2}$: 16 ≤ f ≤ 20) Hz, and beta3 ($\beta_{3}$: 20 ≤ f ≤ 32) Hz. A digital FFT based power spectrum analysis has been applied to quantify the EEG changes, therefore spectral features were computed using the power spectral density of individual channels’ records as determined by Welch Method [99]. A Hamming window was employed to reduce side lobe effect with a frequency ranging from 0.1 to 64 Hz. Power spectral densities were smoothed from segments without overlapping.

Therefore, RP in δ (δRP), RP in θ (θRP), RP in $\alpha_{1}$ and $\alpha_{2}$ ($\alpha_{1}RP$, $\alpha_{2}RP$), RP in $\beta_{1},\;\beta_{2}$ and $\beta_{3}$ ($\beta_{1}RP$, $\beta_{2}RP$, $\beta_{3}RP$), and RP in γ (γRP) can be calculated as in Equation (17) [100]:

(17) $$RP\left(\%\right)=\frac{\sum \mathrm{Used}\;\mathrm{frequency}\;\mathrm{range}\;}{\sum \mathrm{Total}\;\mathrm{range}\;\left(0.1-64\;\mathrm{Hz}\right)}$$

Subsequently, the power ratio of ((δRP/θRP), ($\theta RP/\alpha_{1}RP$), ($\alpha_{1}RP/\alpha_{2}RP$), ($\alpha_{2}RP/\beta_{1}RP$), ($\beta_{1}RP/\beta_{2}RP$), ($\beta_{2}RP/\beta_{3}RP$), ($\beta_{3}RP/\gamma RP$) and (θRP/γRP)) for these spectral potentials were calculated. Additionally, this study is intended to be focused on the markers obtained from EEG in order to detect the changes consequent the stroke-related MCI and VaD during WM task.

## 3. Statistical Analysis

The performance of ANOVA required organization of the denoising results, RP and power ratio results of the 19 channels from the EEG data sets of the three subject groups into five recording sections equivalent to the scalp areas. The post-stroke dementia effects with regard to diminishment of brain activity and complexity can be gleaned from the discrepancies among the scalp areas which can be revealed by regionally averaged characteristics. These regions are frontal (Fp1, Fp2, F3, F4, F7, F8, Fz), temporal (T3, T4, T5, T6), parietal (P3, P4, Pz), occipital (O1, O2), and central (C3, C4, Cz).

The Kolmogorov-Smirnov test was applied as the normality test, Levene’s test produced the homoscedasticity, and Duncan’s test enabled evaluation of the post-hoc comparison. Statistical analysis was performed using SPSS 22.

In the first session of ANOVA was twofold: firstly, one-way ANOVA was conducted to check the performance of the WT, AICA–WT and AICA rejection techniques. The significant differences among the WT, AICA–WT and AICA rejection techniques were evaluated using XCorr as the dependent variable. The significance was set at p ˂ 0.05; Secondly, one-way ANOVA was conducted, a comparative study to check the performance of the AICA–WT proposed technique has been performed. The significant differences among the WT, AICA–WT and AICA rejection techniques were evaluated using PSNR as the dependent variable. The significance was set at p ˂ 0.05.

In the second session of ANOVA, two-way ANOVA was conducted, the group factor (control healthy subjects, stroke-related MCI patients and VaD patients) and scalp regions (frontal, temporal, parietal, occipital and central) were the independent variable and the RP in (δRP, θRP, $\alpha_{1}RP$, $\alpha_{2}RP$, $\beta_{1}RP$, $\beta_{2}RP$, $\beta_{3}RP$, and γRP) was the dependent variable. The significance was set at p ˂ 0.05.

A third session of ANOVA, two-way ANOVA was performed on the power ratios. The group factor (control healthy subjects, stroke-related MCI patients and VaD patients) and the five scalp regions were the independent variable and ((δRP/θRP), ($\theta RP/\alpha_{1}RP$), ($\alpha_{1}RP/\alpha_{2}RP$), ($\alpha_{2}RP/\beta_{1}RP$), ($\beta_{1}RP/\beta_{2}RP$), ($\beta_{2}RP/\beta_{3}RP$), ($\beta_{3}RP/\gamma RP$) and (θRP/γRP)) was the dependent variable. The significance was set at p ˂ 0.05.

## 4. Results and Discussion

### 4.1. Automatic Artifactual Detection

**Figure 5:**
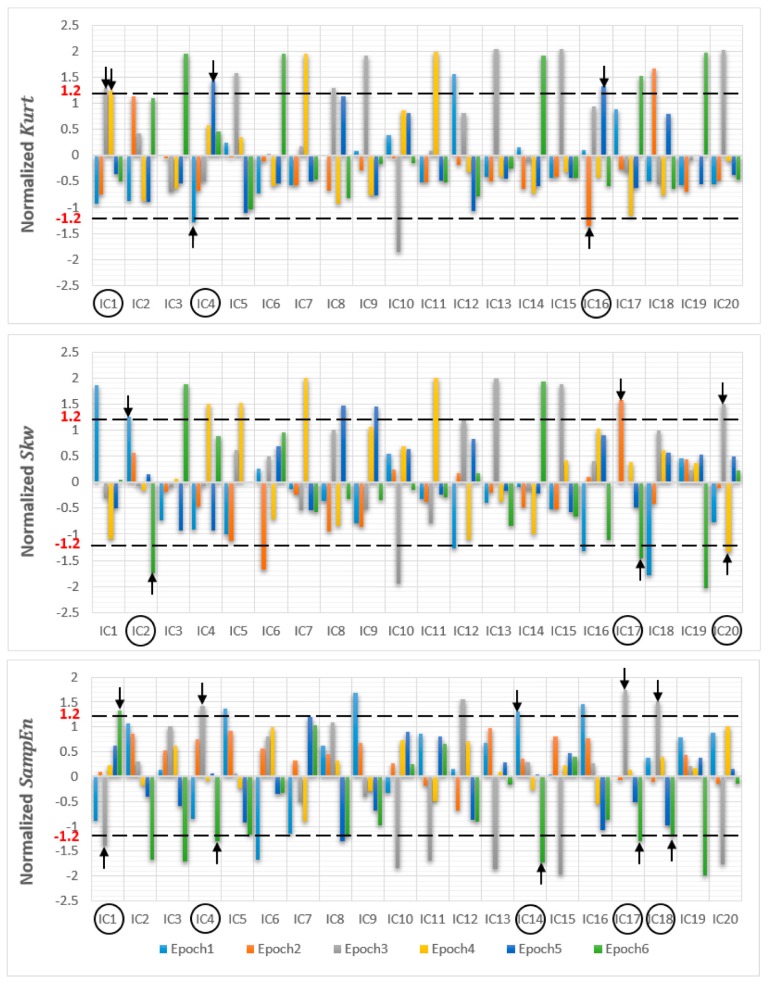
Normalized (kurtosis, skewness and sample entropy) metrics to measure the artifactuality of the Independent components for the first control subject.

**Figure 6:**
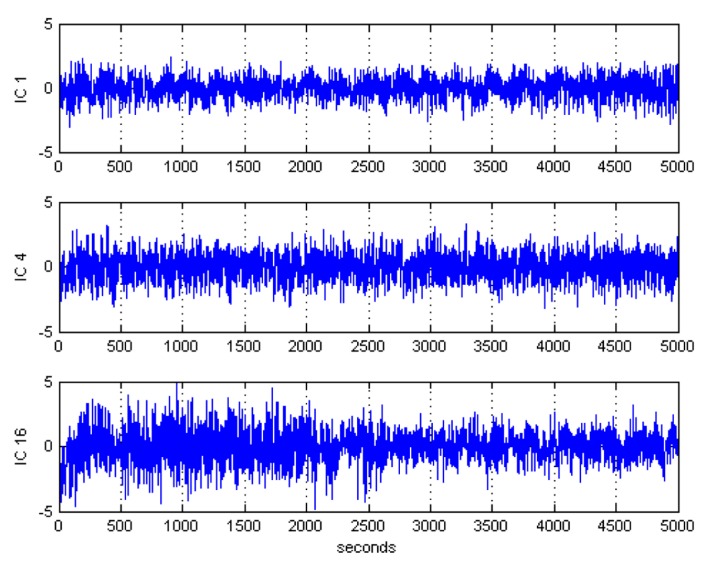
Artifactual components successfully detected by kurtosis for the first control subject.

**Figure 7:**
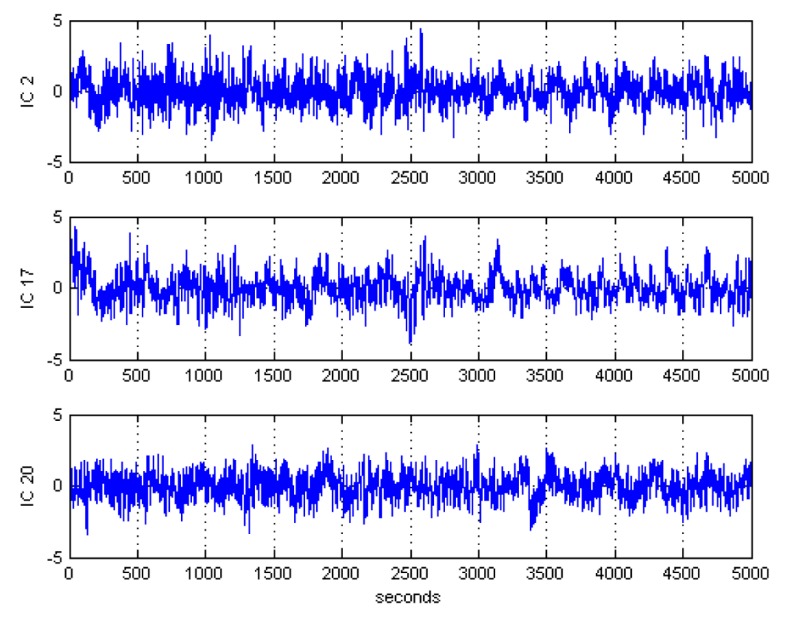
Artifactual components successfully detected by skewness for the first control subject.

**Figure 8:**
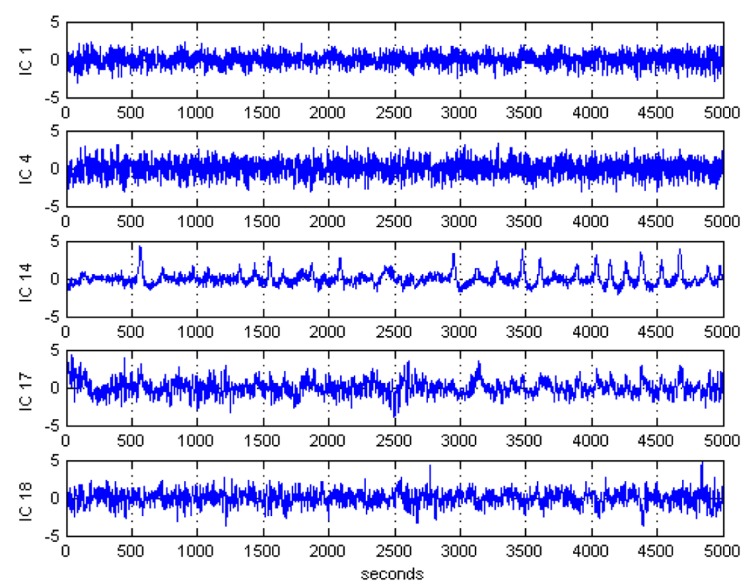
Artifactual components successfully detected by sample entropy for the first control subject.

After ICA decomposition was performed and the estimated ICs were inspected, the artifactuality of the ICs was measured by estimating the three markers: Kurt, Skw, and SampEn. The automatic detection of the estimated ICs using normalized Kurt, Skw, and SampEn metrics to measure the artifactuality of the ICs for the first control subject is shown in Figure 5. The critical selected ICs for the VaD patients, stroke-related MCI patients, and control subjects are summarized in Table 3. In all the analyzed IC epochs, CAs were mainly isolated into only one IC, which had the maximum Kurt and Skw among all the ICs. Our results showed that CAs and OAs were marked by Kurt, Skw, and SampEn because of their amplitude distributions. Kurt and SampEn correctly detected the ICs that explained OAs and MAs. Meanwhile, Skw recognized CAs properly. As in Table 3, an example of artifactual components (ICs) that are successfully detected for the first vascular dementia (VaD) patient using by Kurt, Skw, and SampEn is shown in Figure 6, Figure 7 and Figure 8, respectively.

### 4.2. Denosing Technique Performance Evaluation

**Figure 9:**
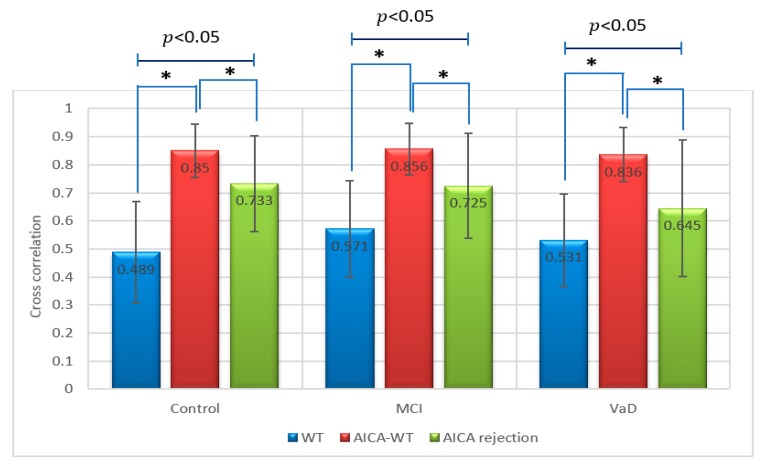
Comparative plot of correlation coefficients between the artifactual EEG signals and the EEG signals after denoising using WT, AICA rejection and AICA–WT techniques for VaD, MCI patients and control subjects. *, indicates a statistically significant difference between groups (p ˂ 0.05).

**Figure 10:**
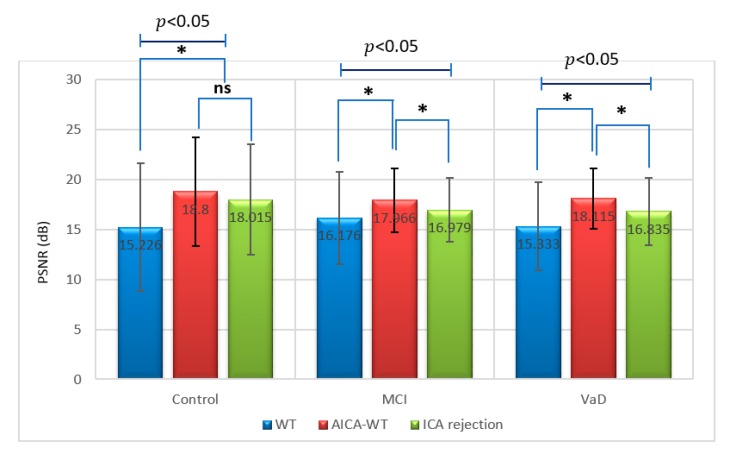
Comparative plot of PSNR between the artifactual EEG signals and the EEG signals after denoising using WT, AICA rejection and AICA–WT techniques for VaD, MCI patients and control subjects. *, indicates a statistically significant difference between groups (p ˂ 0.05). ns means not significant.

The main denoising technique results were summarized statistically by conducting two sessions of one-way ANOVA, one session for the XCorr and the other for PSNR, respectivelly. In these two sessions the descriptive statistics for the XCorr and PSNR were calculated respectivelly between the artifactual EEG signals and the EEG signals after denoising using WT, AICA–WT and AICA rejection techniques for VaD, stroke-related MCI and control subjects. Statistically, the results show that novel AICA–WT technique outperformed the WT denoising and AICA rejection techniques in term of brain activities are largely preserved after artifact removal. Figure 9 and Figure 10 show that the XCorr  and PSNR comparative plots of the proposed AICA–WT technique and the other methods.

Regarding the metric XCorr, there was a statistically significant difference between groups as determined by one-way ANOVA (*p* < 0.05). A Duncan post-hoc test revealed that in control, there was a significant difference between AICA–WT, AICA rejection and WT techniques. In the same manner, in stroke-related MCI, there was a significant difference between AICA–WT, AICA rejection and WT techniques. Finally, in VaD, there was a significant difference between AICA–WT, AICA rejection and WT techniques as well (Figure 9).

On the other hand, a Duncan post-hoc test revealed that, for the PSNR, in controls, there was no significant difference between the AICA–WT and AICA rejection techniques, but these two techniques were significantly different from the WT technique. In contrast, in stroke-related MCI, there was a significant difference between AICA–WT, AICA rejection and WT techniques. Finally, in VaD, there was a significant difference between AICA–WT, AICA rejection and WT techniques as well (Figure 10). Therefore, the proposed novel AICA–WT technique successfully denoised artifactual ICs while preserving nearly all EEG components.

In this manner, the AICA rejection technique, that is, cancelling only the contaminated ICs and the information gathered in the isolated ICs followed by signal reconstruction, may lead to distortions and loss of the underlying cerebral activity and allow for minimum information loss [75].

On the contrary, the WT denoising technique with “sym9” and “SURE” was less effective in reducing noises in the recorded EEG datasets than the AICA–WT denoising and AICA rejection techniques.

Therefore, the proposed AICA–WT denoising technique was used to save the cerebral activities that leaked into ICs. The marked components will not be rejected, but they are arranged in a new dataset in order to denoised using DWT to enhance artifactual ICs. The corrected ICs were returned back to the original set of ICs to be reconstructed and to become a new denoised datasets.

Furthermore, a qualitative review of the reconstructed EEG signals using by an independent skilled expert confirmed that brain activities were largely preserved after using the AICA–WT artifact detection and removal technique. Owing to the wide variety of EEG artifacts, which can be successfully detected and removed using the novel AICA–WT, this technique have been tested on each individual channel of the EEG datasets.

**Figure 11:**
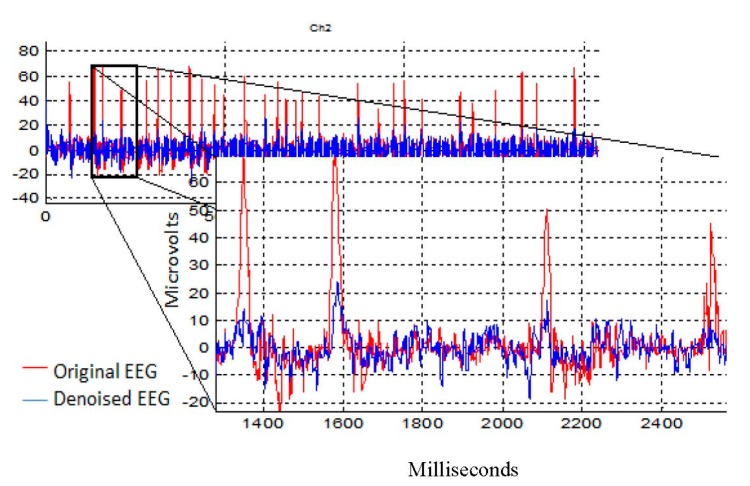
The removal results after the AICA–WT technique was applied on the EEG Ch2 which represents F8 to remove OA.

The artifactual components were sufficiently and successfully suppressed (blue color) compared with the original recorded EEG (red color). As shown in Figure 11, the OAs were successfully suppressed in Ch2 (which represents F8 from the frontal region).

### 4.3. Differences in Spectral Power

The statistical characterizing of the differences in linear spectral distributions among the VaD, stroke-related MCI patients and normal subjects and will be discussed in the following sections.

**Figure 12:**
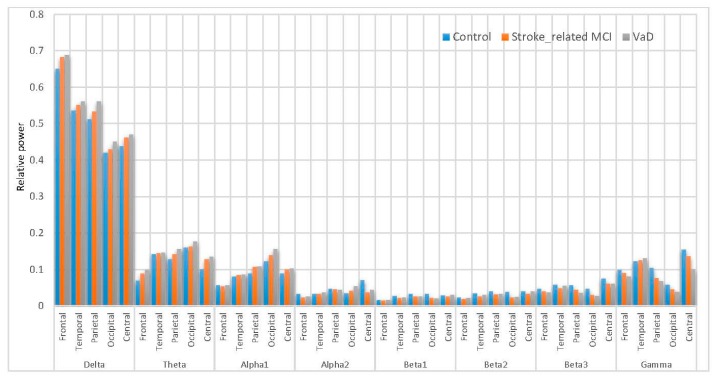
Comparative plot of the relative powers for the five scalp regions of the brain for VaD, Stroke related MCI patients and control subjects without using AICA–WT.

**Figure 13:**
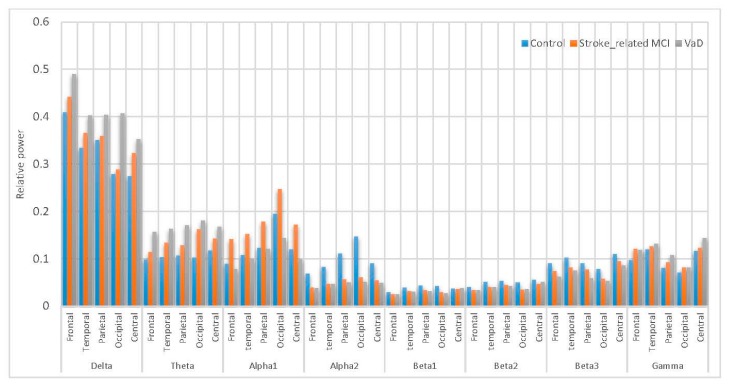
Comparative plot of the RP for the scalp regions for VaD, stroke-related MCI patients and control subjects using AICA–WT.

A comparison of the results with AICA–WT technique with the raw EEG signals (EEG without denoising) has been performed. The significant differences between using the AICA–WT denoising technique and the raw EEG signals in term of improving the spectral power could be shown in Figure 12 and Figure 13, respectively. AICA–WT increases the level of statistical differences between groups then the statistical differences in spectral power between groups in different bands are enhanced more with AICA–WT (ANOVA, p ˂ 0.05) compare to the raw EEG signals (ANOVA, p > 0.05). Thus, the differences in power spectral density distribution among the VaD patients, stroke-related MCI patients, and healthy control subjects will be described based on AICA–WT (ANOVA, p ˂ 0.05).

Figure 13 inspects the slowing down in the spectra of the EEG signals in the VaD, stroke-related MCI patients compare to the normal control healthy subjects. On the one hand, the RP in δ significantly increased (p < 0.05) in VaD and MCI patients compared to healthy subjects and reached the highest values at the frontal and occipital regions ($\delta RP_{VaD}$ > $\delta RP_{MCI}$ > $\delta RP_{Control}$). θRP significantly increased (p < 0.05) in VaD and MCI patients with their highest values at the occipital, parietal, temporal and central regions ($\theta RP_{VaD}$ > $\theta RP_{MCI}$ > $\theta RP_{Control}$). γRP significantly increased (p < 0.05) in VaD and MCI patients to reach the highest values at the central, temporal and frontal regions ($\gamma RP_{VaD}$ > $\gamma RP_{MCI}$ > $\gamma RP_{Control}$). On the other hand, it can also be observed that MCI patients have more power in $\alpha_{1}$ than normal ($\alpha_{1}RP_{MCI}$ > $\alpha_{1}RP_{Control}$ > $\alpha_{1}RP_{VaD}$). $\alpha_{1}RP$ is significantly increase in occipital, parietal and central regions. Notably, $\alpha_{1}RP_{MCI}$ was significantly higher compared with $\alpha_{1}RP_{Control}$. Besides, the differences among these groups are related to the redistribution of power in the sub-bands of $\alpha_{1}$. However, it can also be observed in $\alpha_{2}$ that the VaD and MCI patients having less power compared to the normal subjects ($\alpha_{2}RP_{Control}\;$ > $\alpha_{2}RP_{MCI}$ > $\alpha_{2}RP_{VaD}$). $\alpha_{2}RP$ significant differences were observed in all scalp regions (p < 0.05). The role of βRP activity in WM task can be described as follows: $\beta_{1}RP$ is significantly larger in the reference control subjects compared to VaD and MCI ($\beta_{1}RP_{VaD}$ < $\beta_{1}RP_{MCI}$ < $\beta_{1}RP_{Control}$), significantly in parietal and occipital regions (p < 0.05). $\beta_{2}RP$ is significantly higher in magnitude than $\beta_{1}RP\;$ and larger in control healthy subjects compared to VaD and MCI ($\beta_{2}RP_{VaD}$ < $\beta_{2}RP_{MCI}$ < $\;\beta_{2}RP_{Control}$), significantly in central, parietal and temporal regions. $\beta_{3}RP$ is significantly has the highest magnitude than $\beta_{1}RP$ and $\beta_{2}RP$. $\beta_{3}RP$ is larger in control healthy subjects compared to VaD and MCI ($\beta_{3}RP_{VaD}$ < $\beta_{3}RP_{MCI}$ < $\beta_{3}RP_{Control}$), significantly in central, temporal and parietal regions. Thus, in general cognitive impairment in WM was associated with distributed suppression of alpha activity and with the increase of the theta activity. Moreover, a increment of beta activities were found during task performance. These results can be referred to memory compensation which is related to strategies or processes through which individuals may adapt to, or overcome, decrements or impairments in memory skills. Therefore, these results can be related to a compensation mechanism in MCI patients during memory load and cognitive performance, whereas the control healthy subjects did not have to compensate and the VaD patients could not compensate anymore. Thus, all our findings are consistent with those of other researchers, whose findings showed that the earliest changes in EEG signals among the VaD and MCI patients are related to the increase in δRP, θRP, and γRP activities, as well as the decrease in αRP and βRP activities [5,16,20,21]. Given these results, slowing EEG power among the MCI and VaD patients compared with the healthy control subjects can help recognize MCI and VaD patient activities in post-stroke types of dementia.

**Figure 14:**
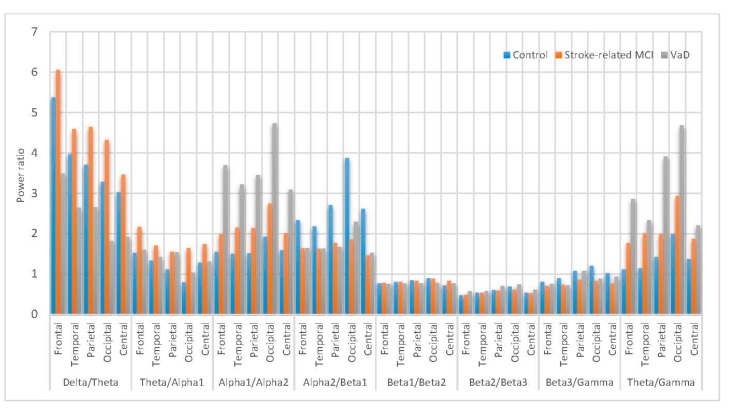
Comparative plot of the power ratios for the scalp regions for VaD, Stroke related MCI patients and control subjects.

Figure 14 shows the power ratio statistical characterization of RP spectral density for the subjects belonging to the different categories (control, MCI and VaD patients). Both (δRP/θRP) and ($\theta RP/\alpha_{1}RP$) of the MCI components are higher compared to the other components. In (δRP/θRP), interestingly MCI components are significantly higher compared to control subjects this is may be due to memory load as  θ is believed to be feasible for cognitive and WM understanding [5], so that in the MCI during WM load and cognitive performance is higher whereas the control subjects did not have to compensate and the VaD patients cannot compensate any more (${\left(\delta RP/\theta RP\right)}_{MCI}$ > ${\left(\delta RP/\theta RP\right)}_{Control}$ > ${\left(\delta RP/\theta RP\right)}_{VaD}$). However, the ($\theta RP/\alpha_{1}RP$) of the MCI components is higher than the other components (${\left(\theta RP/\alpha_{1}RP\right)}_{MCI}$ > ${\left(\theta RP/\alpha_{1}RP\right)}_{VaD}$ > ${\left(\theta RP/\alpha_{1}RP\right)}_{Control}$), but it is insignificantly differentiated among the three groups in all scalp regions. Moreover, ($\alpha_{1}RP/\alpha_{2}RP$) is significantly higher in the VaD patients compared to MCI patients and control subjects $({\left(\alpha_{1}RP/\alpha_{2}RP\right)}_{VaD}$ > ${\left(\alpha_{1}RP/\alpha_{2}RP\right)}_{MCI\;}$ > ${\left(\alpha_{1}RP/\alpha_{2}RP\right)}_{Control}$) in all scalp regions. Notabily, ($\alpha_{2}RP/\beta_{1}RP$) is higher in the control subjects compared to VaD and MCI patients in all scalp regions. ($\beta_{1}RP/\beta_{2}RP$), ($\beta_{2}RP/\beta_{3}RP$) and ($\beta_{3}RP/\gamma RP$) have insignificant differences among the VaD, MCI patients and control subjects which are mainly related to the redistribution of the power in the βRP sub-bands ($\beta_{1}RP,\;\;\beta_{2\;}RP$ and $\beta_{3}RP$) in all scalp regions. Furthermore, (θRP/γRP) is significantly higher in the VaD patients compared to MCI patients and control subjects (${\left(\theta RP/\gamma RP\right)}_{VaD}$ > ${\left(\theta RP/\gamma RP\right)}_{MCI}$ > ${\left(\theta RP/\gamma RP\right)}_{Control}$) in all scalp regions. It can be concluded that the (δRP/θRP) significantly shows slowing in the MCI and VaD patients and could be an indicator for MCI patients, whereas the ($\alpha_{1}RP/\alpha_{2}RP$) and (θRP/γ RP) are markers for VaD detection. Finally, (θRP/γRP) can be a marker for memory decline in VaD and MCI, and increase with the disease severity [101].

To sum up, the (δRP/θRP) could be the reliable index that associated with the MCI detection whereas, the ($\alpha_{1}RP/\alpha_{2}RP$) and (θRP/γRP) ratios could be considered as reliable indices that associated with the VaD identification. So far, these EEG markers might be valuable physiological information that help in improve diagnostic procedure.

## 5. Conclusions

Nineteen channels were used to record the EEG signals during WM. The AICA–WT technique is crucial to remove artifacts and gain insight into dementia by using spectral RP and power ratio features to quantify the changes in the EEG spectra of the stroke-related MCI and VaD patients. The ICA–WT technique was used to denoise the EEG signals of normal subjects, stroke-related MCI, and VaD patients during WM tasks. Spectral analysis was employed to detect abnormalities in the EEG spectra of the three groups’ EEG dataset. The relative powers reflected the slowdown in EEG among the VaD and MCI patients, which resulted in a shift in their power spectrum profiles. An increase in δRP, θRP, and γRP activities, as well as a decrease in αRP and βRP activities were observed. Moreover, the (δRP/θRP) could represent the most sensitive EEG marker of stroke-related MCI detection. Furthermore, ($\alpha_{1}RP/\alpha_{2}RP$) and (θRP/γRP) ratios could be as reliable indices and EEG markers that associated with the VaD identification. The EEG is an appropriate reference in the development of effective treatment for MCI and VaD as its affordability, broad availability, and portability make it a popular clinical screening instrument. In the present study, relative powers and power ratios that could supply relevant diagnostic indexes based on processing of EEG signals were used to analyze the spectra of EEG background activity in subjects with VaD and stroke-related MCI.

## Figures and Tables

**Table 1:** Demographic data of the normal subjects, stroke-related MCI and VaD patients. MMSE and MoCA scores are illustrated as well, (Age in years, MMSE and MoCA scores, mean ± standard deviation SD).

Demographic	Normal	Stroke-Related MCI	VaD
**Number of subjects (Female/Male)**	15 (8/7)	15 (10/5)	5 (2/3)
**Age**	60.06 ± 5.21	60.26 ± 7.77	64.6 ± 4.8
**MMSE**	29.6 ± 0.73	20.2 ± 5.63	14.8 ± 1.92
**MoCA**	29.06 ± 0.88	16.13 ± 5.97	13.2 ± 2.38

**Table 2:** The EEG frequency bands.

Decomposition Levels	EEG Bands	Frequency Range (Hz)	Decomposed Signals
1	Noises	64–128	D1
2	Gamma (γ)	32–64	D2
3	Beta (β)	16–32	D3
4	Alpha (α)	8–16	D4
5	Theta (θ)	4–8	D5
5	Delta (δ)	0–4	A5

**Table 3:** Summary of the artifactual ICs detected by using Kurt Skw and SampEn for the VaD, stroke-related MCI patients and control subjects, (mean ± standard deviation SD).

Subjects	Kurtosis	Skewness	Sample Entropy
**Control**	2.667 ± 1.759	5.133 ± 2.532	4.667 ± 1.633
**MCI**	2.867 ± 1.846	4.6 ± 2.098	>3.867 ± 2.2
**VaD**	3.2 ± 1.6	5.4 ± 2.417	6 ± 1.673
